# Evaluation of a human slow-release buprenorphine formulation in rats – tactile sensitivity and plasma concentration

**DOI:** 10.1186/s42826-026-00293-7

**Published:** 2026-08-24

**Authors:** Spyridoula Kazantzi, Mette N. Jensen, Kristian A. Haanes, Karin M.L. Nordahl

**Affiliations:** 1 Sensory Biology Unit, Translational Research Centre, Copenhagen University Hospital - Rigshospitalet, Glostrup, 2600 Denmark; 2 Section of Cell Biology and Physiology, Department of Biology, University of Copenhagen, Copenhagen, 2200 Denmark; 3 Department of Health Technology, Technical University of Denmark, Kongens Lyngby, 2800 Denmark; 4 Danish Headache Center, Department of Neurology, Copenhagen University Hospital – Rigshospitalet, Glostrup, 2600 Denmark

**Keywords:** Mechanical sensitivity, Analgesia, Sustained-release, Buprenorphine, Rat, Von frey test, Pain-free model, Cephalic allodynia, Human depot formulation

## Abstract

**Background:**

Opioids are considered ideal for managing moderate to severe post-operative pain in laboratory rodents, with buprenorphine being one of the most commonly used analgesics in rats. A long-acting buprenorphine formulation would be highly beneficial to avoid frequent dosing of postoperative animals; however, buprenorphine depot formulations developed for animal use are not available in Europe. The purpose of the present study was therefore to evaluate the effects in rats of a long-acting subcutaneous depot injection with buprenorphine developed for humans (Buvidal).

**Results:**

Buvidal was evaluated in male Sprague Dawley rats alongside conventional short-acting buprenorphine (Temgesic) delivered subcutaneously and orally, a non-opioid analgesic (carprofen), and controls (saline). All three buprenorphine formulations resulted in significantly reduced mechanical sensitivity when assessed in pain-free, naïve rats using the von Frey pressure test at early time points (3–6 h), whereas carprofen and saline had no effect on tactile sensitivity. In a hypersensitivity model of cephalic allodynia, the hypoalgesic effect of Buvidal lasted beyond 24 h. Plasma concentrations support prolonged drug exposure following administration of the long-acting formulation, and levels exceeding 1 ng/mL were observed during periods of reduced tactile sensitivity. No significant effect on locomotor activity was found in an open field test following buprenorphine administration. Transient pica-like chewing behaviour and reduced alertness were observed in some buprenorphine-treated animals during the first hours after drug administration.

**Conclusions:**

The human slow-release buprenorphine formulation Buvidal produced sustained systemic exposure and prolonged hypoalgesic effects in male rats, as demonstrated in the cephalic hypersensitivity model, consistent with depot release kinetics. These results show that Buvidal is pharmacologically active in rats and may represent a potential approach for achieving sustained buprenorphine effects in experimental studies requiring prolonged opioid analgesia where rodent-specific formulations are unavailable, although further evaluation of safety, dosing, and generalizability is required.

**Supplementary Information:**

The online version contains supplementary material available at 10.1186/s42826-026-00293-7.

## Background

In the post-operative care of laboratory rodents, opioids, non-steroidal anti-inflammatory drugs (NSAIDs), and local analgesics are the mainstay in analgesic treatment [1]. Buprenorphine, a partial agonist at the µ-opioid receptor [2], has a strong analgesic effect comparable to that of full µ-receptor agonists [3–5], with a rapid onset and a longer duration of action compared to morphine [4, 6]. Side effects such as pica, hyperactivity, sedation, and reduced food intake have been reported in rodents [7–11]. However, in contrast to many other µ-receptor agonists, the adverse effects of buprenorphine appear to exhibit a ceiling effect, suggesting a broader safety margin [12–15]. These advantages make buprenorphine an excellent option when opioids are warranted, and accordingly, it is one of the most commonly used post-operative analgesics in laboratory mice and rats [16]. Nevertheless, recommended dosing intervals for subcutaneous buprenorphine remain relatively short, every six to eight hours [6], making long-term treatment laborious and potentially stressful for the animals [9]. This stress can be reduced by voluntary oral administration [17–19], and administering buprenorphine in a tasty nut paste or in food pellets have proven easy and effective in achieving pain relief [20, 21]. However, frequent administrations may still be required, and as with subcutaneous delivery, this increases the risk of insufficient analgetic coverage in between doses. With voluntary oral ingestion, there is also a risk that decreased appetite or poor palatability of the mixture may reduce drug intake and, consequently, compromise analgesic coverage.

A long-acting buprenorphine formulation would be highly beneficial to avoid frequent dosing of post-operative animals, and to obtain a more stable drug concentration over time. Slow-release depot formulations of buprenorphine for rodents are available in the United States, Buprenorphine Base Lab ER (Wedgewood Pharmacy, NJ, USA) and Ethiqa XR (Fidelis Animal Health Inc., NJ, USA), which both have shown good results with analgesic effects lasting up to 72 h in rats [22–25]. Unfortunately, depot formulations of buprenorphine developed for animal use are not available in Europe. This has spurred others to develop their own in-house slow-release preparations [9, 26, 27]. Commercially there are options for human use available in the EU, intended for treating opioid addiction. The purpose of the present study was therefore to evaluate the effect of a long-acting subcutaneous depot injection with buprenorphine available for humans (Buvidal, Camurus, Lund, Sweden) on tactile sensitivity, as an indicator of analgesic activity, in rats. Standard “short-acting” immediate-release buprenorphine was used as positive control.

Buvidal was evaluated in both pain-free, naïve rats without surgery or injury and in a hypersensitive rat model of cephalic allodynia induced by dural inflammation. A hypersensitivity model was chosen to allow inclusion of vehicle-treated controls without inducing significant pain. Mechanical sensitivity was selected as the primary endpoint for assessing opioid-induced effects, as buprenorphine’s antinociceptive responses have been reported to be less robust in thermal withdrawal assays (e.g., hot plate, hot water, or laser) [20, 28, 29]. In addition, others have demonstrated that buprenorphine increases mechanical withdrawal thresholds in healthy Sprague Dawley rats when assessed using a plantar paw pressure device [30], supporting the use of mechanical sensitivity assays in pain-free animals. Accordingly, the electronic von Frey test was used to evaluate opioid effects on mechanical sensitivity in both hypersensitive and naïve animals. Analgesiometric testing was complemented with measurements of plasma concentrations and assessment of locomotor activity following opioid treatment.

## Methods

### Animals

Experiments were performed using male Sprague Dawley (SD) rats (NTac: SD, Taconic Biosciences, Ejby, Denmark). Housing conditions are detailed in Supplementary materials. Stratified randomization using the RAND() function in Microsoft Excel, based on body weight, was performed across cages. All experiments were approved by the Danish Animal Experiments Council (License: 2023-15-0201-01469 and 2020-15-0201-00751) in compliance with European Directive 2010/63/EU.

### Drugs

Slow-release buprenorphine (SR-BUP) (Buvidal, 8 mg. Camurus, Lund, Sweden), delivered in a single human dose syringe, was dispensed into a sterile Eppendorf tube, 50 mg/mL (8 mg/0.16 mL [lowest concentration available]). A 0.3 mL insulin syringe was then used to withdraw and administer 10 µL (0.5 mg)/rat s.c. corresponding to approximately 1.1–1.6 mg/kg. Depot formulations are not suitable for dilution, as that may affect the rate of release, cause specific interactions, or affect the stability of the drug. Furthermore, the homogeneity of the preparation might change following dilution, making dosing difficult. Therefore, we chose not to attempt to dilute Buvidal, and the dose administered thus represents the lowest practically achievable dose using this formulation.

Immediate-release buprenorphine (IR-BUP) (Temgesic 0.3 mg/mL. Indivior, Dublin, Ireland) diluted in 0.9% saline to 0.1 mg/mL was administered subcutaneously (s.c.) at 1 mL/kg resulting in a dose of 0.1 mg/kg. This corresponds to the upper end of the dosing range recommended in our facility for rats (0.05–0.1 mg/mL), which is based on guidance from the Danish Animal Experiments Inspectorate 2014 report [31].

Oral buprenorphine (PO-BUP) tablets (Temgesic sublingual tablets, 0.2 mg. Indivior, Dublin, Ireland) were crushed to a powder and mixed with a hazelnut spread (Nutella^®^, Ferrero, Pino Torinese, Italy) to a 0.2 mg/g mixture and administered individually (over 2–10 min) to the rats (in a separate cage) at 2 g/kg corresponding to 0.4 mg/kg [32]. For equality, and to habituate the animals to the taste all rats were given neutral un-spiked Nutella in their home cage once daily from day − 5 until study end. PO-BUP was included as this is a common analgesic strategy at our facility.

Carprofen (CAP) (Rimadyl Bovis vet, 50 mg/mL. Zoetis Animal Health, Copenhagen, Denmark), an NSAID, was included as a non-opioid control widely used for postoperative analgesia in rodents. It was diluted in sterile water to 5 mg/mL and administered at 1 mL/kg s.c., corresponding to 5 mg/kg.

Saline (9 mg/mL NaCl) was used as negative control and administered s.c. at 1 mL/kg. Rats receiving oral buprenorphine were also given a saline injection to control for the possible effect of any stress experienced during restraint and injection.

### Dural inflammation model

Dural inflammation was induced as previously described to generate a model of cephalic allodynia [33, 34]. In short, rats were anesthetized with isoflurane (1–2 vol% isoflurane [5 vol% during induction], air 1 L/min, O_2_ 0.3 L/min), and the skin over the crown of the head was incised to expose the skull. A 3 × 3 mm opening was then drilled in the skull to expose the underlying dura mater. Complete Freund’s adjuvant (F5881, Sigma-Aldrich, Taufkirchen, Germany) was applied to the dura for 15 min before closing the opening with bone wax and suturing the skin. Carprofen (5 mg/kg) was administered s.c. perioperatively and again 24 h after surgery. The rats were monitored daily for clinical signs incompatible with the expected light-to-moderate headache-like phenotype of the model, such as severely affected general condition or abnormal stereotypic behaviours (e.g. circling), which constituted humane endpoints and would lead to study exclusion and euthanasia.

### Measurement of tactile sensitivity

Mechanical sensitivity was evaluated before and after drug administration using the von Frey pressure test [35]. Measurements were obtained with a hand-held electronic von Frey device (IITC Life Science, Los Angeles, CA, USA). Plantar (right hind paw for consistency) and periorbital pressure withdrawal thresholds were determined as previously described [30]. The filament was applied with an increasing force up to 400 g or until the rat withdrew and/or vocalized. The withdrawal threshold was calculated as the average pressure measurement of four pressure points between the footpads (plantar threshold) or three pressure points on the forehead (periorbital). The operator was blinded to treatment allocation. Non-responsiveness during baseline von Frey testing or the presence of baseline tactile allodynia (mean threshold < 150 g) constituted exclusion criteria.

### Plasma concentration

Blood samples were collected via a tail vein with the rat under general isoflurane inhalation anaesthesia. Plasma was prepared by gently mixing whole blood with 3.2% (0.109 M) sodium citrate at a ratio of 1:9 (10%v/v) and centrifuged at 4000 g for 5 min. Buprenorphine concentration was measured using a buprenorphine ELISA kit (Neogen Ireland, Wicklow, Ireland) according to the manufacturer’s instructions and with an added buprenorphine standard curve, similar to what others have done [36]. The standard was produced by diluting 0.3 mg/mL buprenorphine (Temgesic) in saline and finally in naïve rat plasma to a concentration of 10 ng/mL. This was then serially diluted 2-fold in naïve plasma to yield an eight-point standard curve with the following concentrations: 10, 5, 2.5, 1.25, 0.625, 0.313, 0.156, and 0.078 ng/mL. A blank sample consisting of un-spiked naïve plasma was also included. Plasma samples were first added undiluted. Samples with OD values at or above the highest standard (10 ng/mL) were later diluted 1:5 and 1:20 in naïve plasma pool and re-run on a subsequent plate. All samples and controls were run in duplicates. Measured concentrations were interpolated from the standard curve included on each plate and diluted samples were multiplied by the dilution factor to obtain back-calculated original concentrations. A therapeutic threshold of 1 ng/mL for buprenorphine in plasma has been widely adopted in preclinical research [22, 26, 36, 37], with some studies suggesting it aligns with clinical observations [38]. However, definitive evidence confirming this value as the minimum effective concentration remains limited. For the sake of consistency with existing literature, we will adopt this value, while acknowledging the possibility that the actual therapeutic threshold may differ.

### Locomotor activity

To assess locomotor activity an open field test (OFT) was performed. A plexiglass box measuring 50 × 50 cm and 34 cm high with black opaque sides and no lid was used as testing arena. Ambient light was dimmed to 10 lx measured in the middle of the box using a Mini Light meter (UT383, Uni-Trend Technology, Guangdong, China). Following drug injection, the animals were allowed to rest in their home cage for 20 min, they were then placed individually in the middle of the OFT arena and activity was tracked for 20 min using a camera and video tracking software (ANY-maze video tracking system v.7.48, Stoelting Co., Wood Dale, IL, USA). Rats were tested in a random cage-wise fashion, the operator was not blinded to treatment allocation but had no influence on the tracking results. The box was wiped clean in-between rats. For the 24 h measurement on the second day the rats were placed individually in the test box and the test was performed as described above. Total distance travelled and time spent mobile were assessed. Immobility was registered as soon as the animal had been inactive for minimum 5 s.

### Study design

*Part 1*: To assess the effects of SR-BUP on mechanical sensitivity relative to standard in-house analgesic regiments, 66 rats were randomly assigned to one of five treatments: SR-BUP (*n* = 18), IR-BUP (*n* = 12), PO-BUP (*n* = 12), the non-opioid control CAP (*n* = 12), or saline (*n* = 12). Within the SR-BUP group, six animals received Buvidal from a vial that had been opened previously and stored for three weeks at room temperature protected from light prior to administration. All treatments were given at time 0. Injections were delivered s.c. in the lateral flank region. Pressure threshold assessment using the von Frey test was performed before treatment (baseline, all rats) and after dosing at 3 or 6 h, and again at 24 h and/or 30 h (*n* = 6/time-point/group). A maximum of three assessments within 24 h per animal was used to maintain reliability, as more frequent testing may induce learning effects. Time-points were selected based on the reported duration of action of IR-BUP, to capture early (3 h), midway (6 h), and later (24 h) post-treatment responses, with an additional 30 h time-point included to assess effects beyond 24 h. Group sizes (*n* = 6/time-point) were determined by power calculation, assuming a minimum relevant difference of 60 g (twice the standard deviation) between group means, a standard deviation of 30 g based on von Frey data from a pilot IR-BUP treatment study, significance level (α) of 0.05, and power (1 − β) of 0.8.

*Part 2*: To assess plasma concentrations of Buvidal, and compare with standard IR-BUP treatment, a new set of 20 rats were randomly assigned to receive either SR-BUP (*n* = 10) or IR-BUP (*n* = 10). Four blood samples were collected from each rat (< 5% of total blood volume was collected in total) to measure plasma buprenorphine concentration over time following a single s.c. injection: before treatment (baseline, all rats), and at 3 h, 24 h, and 30 h for the SR-BUP group, and at 3 h, 6 h, and 24 h for the IR-BUP group. No saline or PO-BUP groups were included, as the main comparator to Buvidal was IR-BUP. Time-points were chosen to match von Frey testing. Following a five-week washout period the rats were randomly assigned to a new treatment group: saline (*n* = 6), IR-BUP (*n* = 7), or SR-BUP (*n* = 7). Following the injection, activity assessment was performed at 20 min and 24 h after treatment using the OFT.

Part 3: To evaluate the effects of Buvidal in a hypersensitive condition, 16 rats were subjected to dural inflammation surgery followed by s.c. SR-BUP (n=8) or saline control (n=8) treatment. Periorbital (local sensitivity) and plantar (global sensitivity) von Frey were performed before surgery (Baseline), after surgery and one day before treatment (Sensitized dura), and at 3h, 24h, and 30h after treatment. Injections (time 0h) were performed 11-12 days after surgery to allow surgical recovery before opioid evaluation while retaining dural sensitivity. Eight rats were included per group to ensure sufficient numbers for von Frey testing in case early euthanasia became necessary for some rats due to humane endpoints being reached following surgery.

All rats were monitored closely, particularly the first hours after drug administration, and then once daily for the following four days. The injection site was inspected for signs of skin irritation. Behaviour and activity were also evaluated, looking for signs of distress or overdose. Rats were weighted at study start and again if prompted by signs of discomfort lasting > 24 h. Humane endpoints were set to > 15% weight loss from baseline, respiratory compromise, and/or persistent sedation. At the end of the observation period the animals were euthanized by decapitation under deep carbon dioxide anaesthesia.

Gross injection site reactions were not seen, nor was respiratory distress, or persistent sedation. No rats were excluded from the study.

### Statistics

Statistical comparisons were performed using repeated measures two-way ANOVA or mixed effects model, followed by Dunnett’s or Sidak’s multiple comparisons test comparing within group effects over time or between groups effects at each time-point. For comparing means to a clinically relevant threshold One sample t-tests were used. All data sets passed the Shapiro-Wilk normality test. Statistical significance was defined as *p* < 0.05. In the main text, p-values are reported to three decimal places, with values below 0.001 indicated as *p* < 0.001. Data analysis and result visualization were performed using GraphPad Prism (version 10.1.2, GraphPad Software, La Jolla, CA, USA). All reported statistics, including exact p-values, are presented in Suppl. Table S1.

## Results

### Human slow-release buprenorphine reduces tactile sensitivity in pain-free rats

Plantar mechanical sensitivity was assessed prior to and after analgesic or saline treatment in healthy rats. Statistical analysis showed significant main effects of both time-point (F(4, 130) = 40.48, *p* < 0.001) and treatment (F(4, 55) = 10.61, *p* < 0.001), qualified by a significant interaction between the two factors (F(16, 130) = 7.25, *p* < 0.001), indicating that treatment groups responded differently over time. Subsequent multiple testing showed that time had a significant effect only in the three buprenorphine groups.

**Fig. 1:**
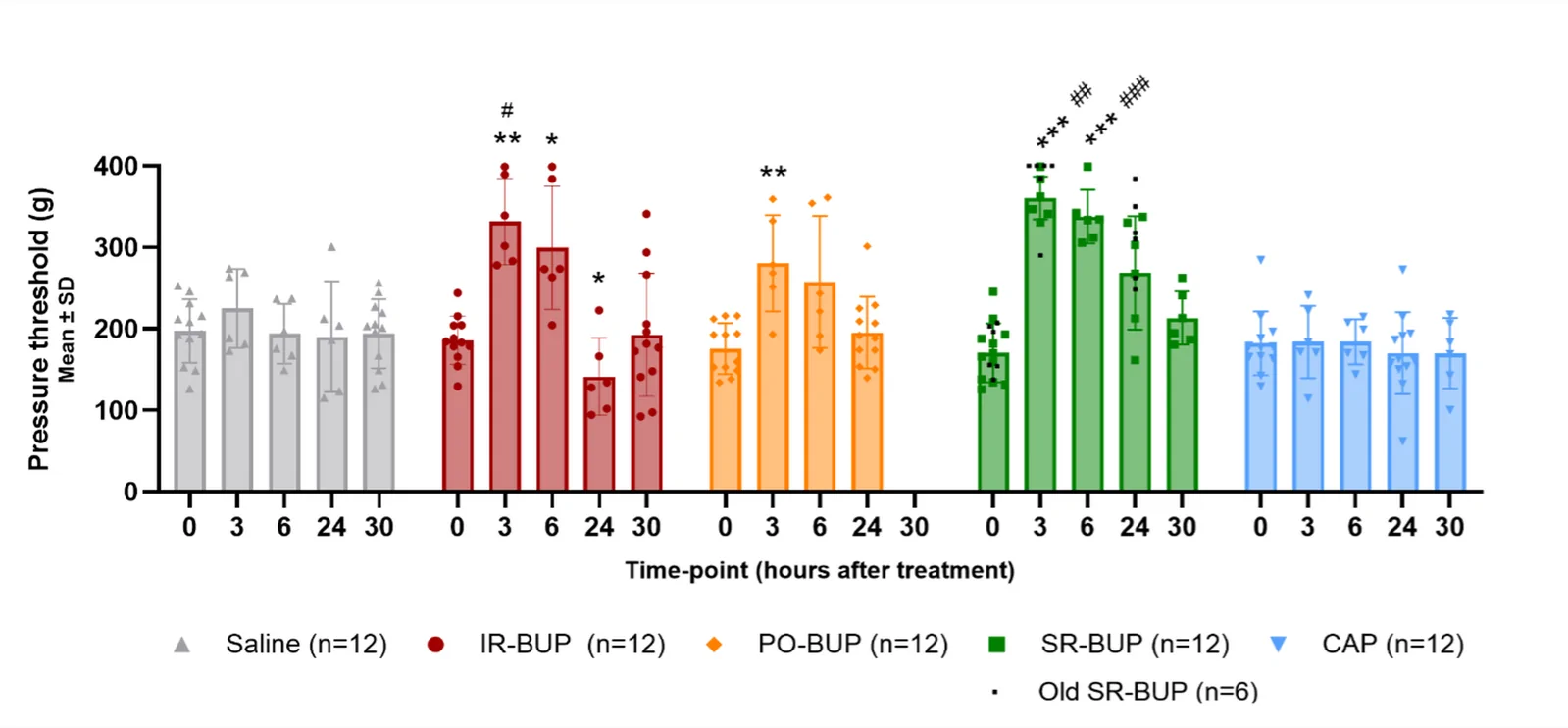
Tactile sensitivity following buprenorphine treatment. Male Sprague Dawley rats received a single administration of either saline (1 mL/kg s.c.), IR-BUP (0.1 mg/kg s.c), PO-BUP (0.4 mg/kg p.o), the depot formulated SR-BUP (~ 1.5 mg/kg s.c), or the non-opioid control CAP (5 mg/kg s.c.). Plantar withdrawal thresholds, assessed using mechanical von Frey testing prior to treatment (0 h) and at different time-points after treatment, are presented for each administration group. Exploratory data from rats receiving SR-BUP from a previously opened vial (Old SR-BUP, *n* = 6) are shown as small black symbols for transparency but were not included in the calculation of mean thresholds or in the statistical comparisons. Statistical comparisons were performed using repeated measures two-way ANOVA followed by Dunnett’s multiple comparisons test within groups comparing each time-point to baseline (*; *p* < 0.05, **; *p* < 0.01, ***; *p* < 0.001), and between groups comparing treatment to saline at each time-point (#; *p* < 0.05, ##; *p* < 0.01, ###; *p* < 0.001). Only significant (*p* < 0.05) differences are shown. CAP; carprofen, IR-BUP; immediate-release buprenorphine, PO-BUP; oral buprenorphine, p.o; *per os* (orally), s.c; subcutaneous, SR-BUP; slow-release buprenorphine

IR-BUP treatment significantly increased withdrawal thresholds (reduced tactile sensitivity) compared to baseline (187 ± 30 g) at the 3 h (333 ± 53 g, *p* = 0.003) and 6 h (301 ± 76 g, *p* = 0.039) time-points (Fig. 1). In contrast, a significant decrease in pressure thresholds, indicating increased sensitivity, was seen at 24 h post treatment (143 ± 47 g, *p* = 0.026). Comparisons were also made with saline-treated animals at each time-point, and a significant threshold difference was seen at the 3 h time-point (*p* = 0.015). PO-BUP significantly reduced mechanical sensitivity at 3 h (282 ± 59 g, *p* = 0.001), but not at 6 h (259 ± 81 g, *p* = 0.217), when compared with pre-treatment levels (177 ± 31 g). Carprofen and saline had no significant effect on mechanical sensitivity at any time-point (Fig. 1).

The SR-BUP formulation was delivered at a fixed dose per rat resulting in an average dose of 1.6 mg/kg. Treatment with SR-BUP reduced mechanical sensitivity, showing significantly increased pressure thresholds at 3 h (371 ± 36 g, *p* < 0.001) and 6 h (339 ± 33 g, *p* < 0.001) compared with pre-treatment values (173 ± 33 g) (Fig. 1). Thresholds were no longer significantly increased at 24 h (291 ± 62 g, *p* = 0.058), although a similar trend was observed, and declined further at 30 h (214 ± 33 g, *p* = 0.761). When compared with saline at each time-point, SR-BUP-treated animals showed significantly higher withdrawal thresholds at 3 h (*p* = 0.001) and 6 h (*p* < 0.001) (Fig. 1). The cohort receiving SR-BUP from a previously opened vial was excluded from the primary statistical analysis to preserve a balanced group design, however, exploratory comparison suggested a similar response profile between animals receiving SR-BUP from newly opened and previously opened vials.

### SR-BUP results in sustained plasma buprenorphine concentrations compared with IR-BUP

**Fig. 2:**
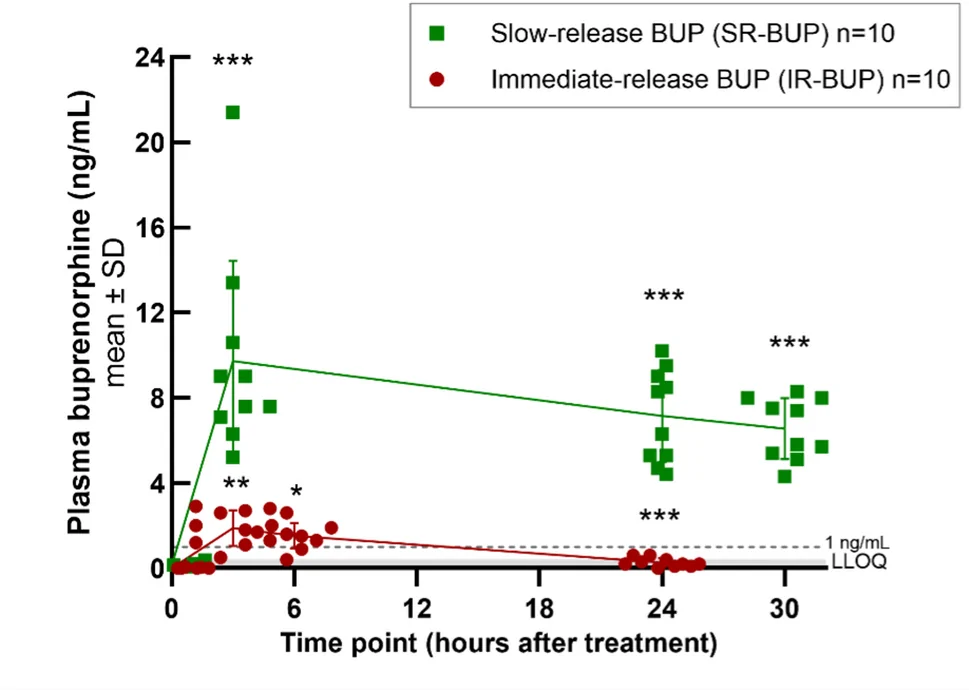
Plasma buprenorphine concentration following a single subcutaneous administration. Male Sprague Dawley rats received a single subcutaneous injection of either IR-BUP (0.1 mg/kg) or the depot formulated SR-BUP (~ 1.5 mg/kg). Buprenorphine concentration was measured in plasma samples collected before treatment (time 0) and at different time-points after drug administration. Buprenorphine concentrations are presented, showing individual values and mean with standard deviation at each time-point. Clinically significant concentration of 1 ng/mL is marked with a dotted line. The area below LLOQ (0.31 ng/mL) is shaded. One sample t-tests were used for statistical comparisons within groups between the 1 ng/mL threshold and each time-point after baseline (*; *p* < 0.05, **; *p* < 0.01, ***; *p* < 0.001). IR-BUP; immediate-release buprenorphine, LLOQ; lower limit of quantification, SR-BUP; slow-release buprenorphine

Buprenorphine concentration in plasma was measured before drug delivery (baseline) and again at different time-points after IR-BUP or SR-BUP administration. The average administered SR-BUP dose was 1.5 mg/kg. The lower limit of quantification (LLOQ) was defined as 0.31 ng/mL, corresponding to the lowest standard that consistently produced a signal distinguishable from the blank with acceptable precision (intra-plate %CV < 5%). IR-BUP concentration was significantly above the clinically relevant threshold of 1 ng/mL at the 3 h (1.9 ± 0.8 ng/mL, *p* = 0.009) and 6 h (1.5 ± 0.6 ng/mL, *p* = 0.024) time-points (Fig. 2), corresponding to the time-points when there was a decrease in tactile sensitivity. At 24 h the concentration had fallen significantly below 1 ng/mL (0.3 ± 0.2 ng/mL, *p* < 0.001). Thirteen SR-BUP samples were at or above the assay upper limit (10 ng/mL) and were therefore diluted and re-analysed. Plasma buprenorphine concentrations in the SR-BUP-treated group were significantly above 1 ng/mL at all three post-treatment time-points (3 h: 9.7 ± 4.7 ng/mL, *p* < 0.001, 6 h: 7.2 ± 2.1 ng/mL, *p* < 0.001, and 24 h: 6.6 ± 1.4 ng/mL, *p* < 0.001) (Fig. 2), consistent with the long-lasting effect seen in the von Frey test.

### Hyperactivity was not observed following buprenorphine treatment

**Fig. 3:**
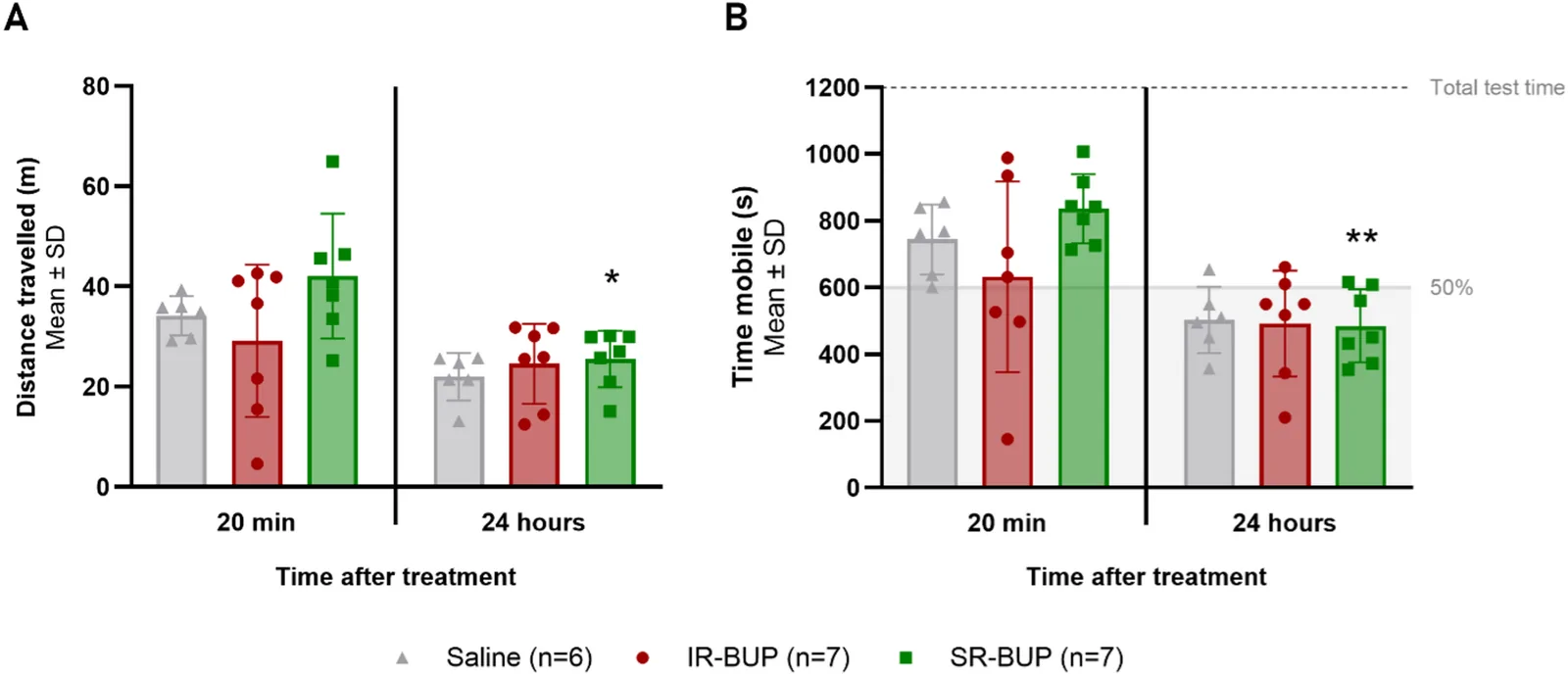
The effect of buprenorphine on locomotor activity. Male Sprague Dawley rats received a single subcutaneous injection of either saline, IR-BUP (0.1 mg/kg), or the depot formulated SR-BUP (~ 1.1 mg/kg). Open field test was performed at 20 min following treatment and again at 24 h. Total distance travelled **(A)** and total time spent mobile **(B)** at the two time-points are presented for the three treatment groups. Statistical comparisons were made using repeated measures two-way ANOVAs followed by Sidak’s multiple comparisons test, comparing the effect of treatment across time for both parameters evaluated (*; *p* < 0.05, **; *p* < 0.01). IR-BUP; immediate-release buprenorphine, SR-BUP; slow-release buprenorphine

Opioid treatment has been reported to induce hyperactivity in mice, and slow-release preparations may be particularly prone to have this side effect [9]. Therefore, locomotor activity was assessed following SR-BUP and IR-BUP treatment. Distance travelled and time spent mobile were evaluated during a 20 min OFT performed 20 min and 24 h after s.c. buprenorphine or saline administration. SR-BUP was delivered at 10 µL/rat resulting in an average dose of 1.1 mg/kg. Statistical analysis showed significant main effects of time-point on both distance travelled (F(1, 17) = 11.65, *p* = 0.003) and time spent mobile (F(1, 17) = 17.42, *p* = 0.001), but no treatment effect or interaction between the two factors for either parameter, indicating that there was a similar trend in reduced locomotor activity with time regardless of treatment. Post tests confirmed the time effect within the SR-BUP group only, where both distance travelled (*p* = 0.023) and time spent mobile (*p* = 0.007) decreased from the 20 min test to the 24 h test (Fig. 3). These within-group results are however interpreted cautiously because the main test showed no interaction.

During routine monitoring across all studies, with rats housed in mixed-treatment cages, signs of hyperactivity were not observed. On the contrary, several buprenorphine-treated rats, particularly those treated with IR-BUP, displayed a transient, almost docile behaviour in their home cages approximately 0.5–3 h after drug administration. This was characterized by reduced alertness and a tendency to sit while slowly swaying their heads from side to side. Pica-like behaviour, observed as chewing on bedding material, was noted in some IR-BUP and SR-BUP treated rats, but not in PO-BUP treated rats, during this period. These behaviours were not observed at the 24 h time-point.

### SR-BUP produces sustained effects in a hypersensitivity model

**Fig. 4:**
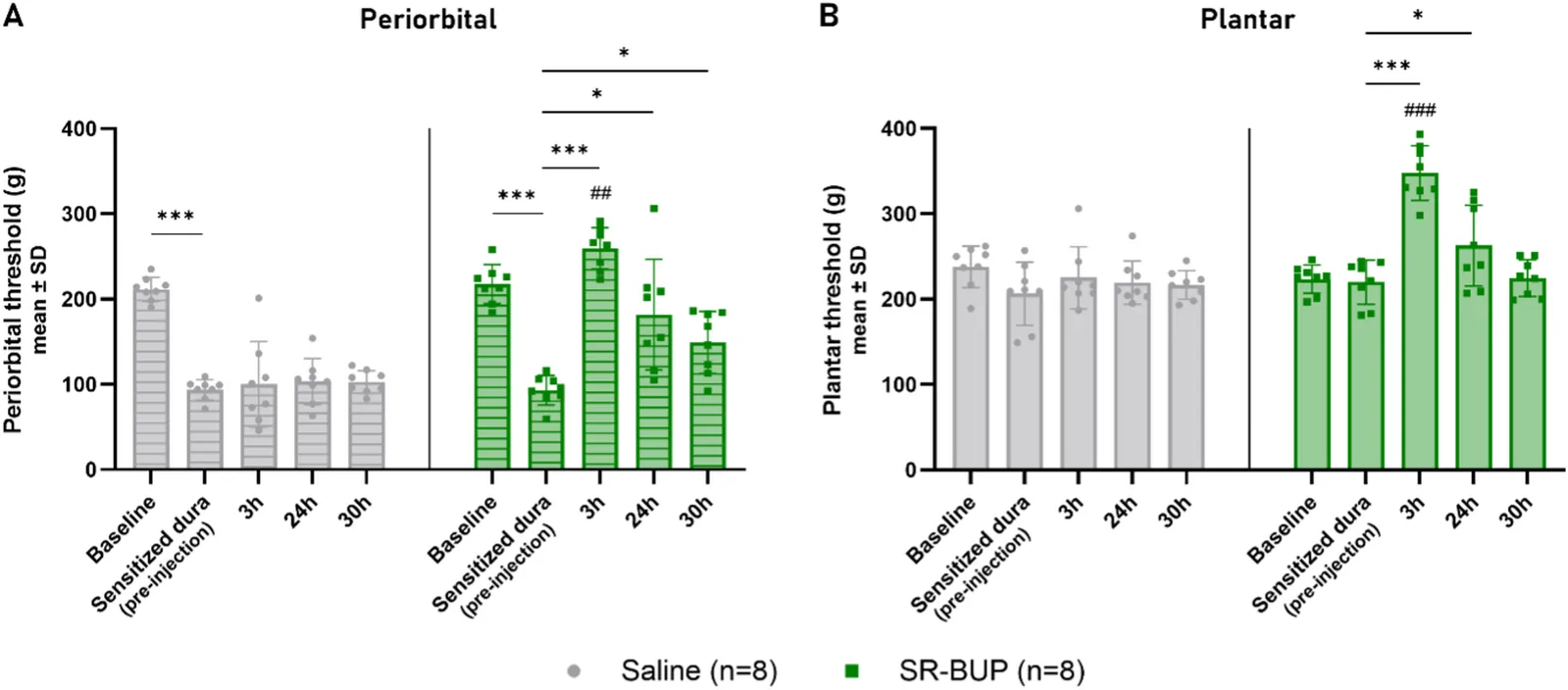
Tactile sensitivity following buprenorphine treatment in dural inflammation model. Male Sprague Dawley rats received a single injection of either ~ 1.3 mg/kg SR-BUP (*n* = 8) or saline (*n* = 8) following induction of dural inflammation in a cephalic hypersensitivity model. Periorbital **(A)** and plantar **(B)** withdrawal thresholds prior to inflammatory surgery (Baseline), after surgery and before treatment (Sensitized dura, pre-injection), and at different time-points after treatment (3 h, 24 h, 30 h) are presented for both treatment groups. Statistical comparisons were performed using two-way ANOVA followed by Dunnett’s multiple comparisons test comparing each time-point with the pre-injection threshold (*; *p* < 0.05, ***; *p* < 0.001). Planned comparisons between baseline and the 3 h time-point were performed using t-tests for SR-BUP-treated animals in both periorbital and plantar datasets (##; *p* < 0.01, ###; *p* < 0.001). Only significant (*p* < 0.05) differences are shown. SR-BUP; slow-release buprenorphine

In the hypersensitive dural inflammation model, periorbital mechanical sensitivity was significantly affected by both treatment (F(1, 14) = 25.87, *p* < 0.001) and time (F(2.76, 38.84) = 51.53, *p* < 0.001), qualified by a significant treatment x time interaction (F(2.76, 38.61) = 24.61, *p* < 0.001), indicating that saline- and SR-BUP-treated animals responded differently over time. Post hoc analysis confirmed that, prior to treatment, both groups displayed marked mechanical hypersensitivity following dural inflammation, as withdrawal thresholds at the pre-injection (post-surgery) time-point were significantly reduced compared to baseline (saline: 94 ± 12 g vs. 212 ± 14 g, *p* < 0.001; SR-BUP: 93 ± 17 g vs. 218 ± 23 g, *p* < 0.001), consistent with a headache-like pain state induced by the model (Fig. 4.A). Following saline administration, withdrawal thresholds remained at pre-injection (sensitized dura) levels at all subsequent time points (3 h: 100 ± 50 g, 24 h: 103 ± 27 g, and 30 h: 103 ± 13 g), indicating no observable effect of the control treatment on mechanical sensitivity. In contrast, SR-BUP-treated animals exhibited significantly increased withdrawal thresholds at 3 h (260 ± 24 g, *p* < 0.001), 24 h (182 ± 65 g, *p* = 0.019), and 30 h (149 ± 36 g, *p* = 0.015) relative to the pre-injection value, consistent with sustained analgesic activity. A planned comparison further showed that thresholds at 3 h exceeded baseline levels (*p* = 0.002), demonstrating pronounced opioid-induced hypoalgesia (Fig. 4.A).

Plantar mechanical sensitivity was also assessed. Here, significant effects of treatment (F(1, 14) = 15.27, *p* = 0.002) and time (F(3.29, 46.09) = 18.52, *p* < 0.001) were observed, qualified buy a significant interaction (F(3.29, 46.09) = 15.84, *p* < 0.001), indicating different responses between the treatment groups over time. Post hoc analysis showed no significant effect of dural inflammation on plantar sensitivity in either group, and thresholds in saline-treated animals remained stable throughout the experiment. In contrast, SR-BUP treatment significantly increased withdrawal thresholds at 3 h (348 ± 32 g, *p* < 0.001) relative to the pre-injection (sensitized dura) value (224 ± 17 g), and this effect persisted at 24 h (263 ± 47 g, *p* = 0.047) (Fig. 4.B). A planned comparison between baseline and the 3 h time-point further confirmed the SR-BUP-induced increase in plantar withdrawal thresholds (*p* < 0.001) (Fig. 4.B). The average SR-BUP dose delivered was approximately 1.3 mg/kg.

## Discussion

A slow-release buprenorphine preparation intended for human use was evaluated in male SD rats. Tactile sensitivity was assessed as well as locomotor activity, and plasma drug levels were measured over time.

All three buprenorphine formulations tested (SR-BUP, IR-BUP, and PO-BUP) significantly reduced mechanical sensitivity in naïve rats, as reflected by increased withdrawal thresholds in the von Frey test. Our results demonstrate that the effects of buprenorphine on mechanical sensitivity can be evaluated in an uninjured pain-free setting. The transient lethargy and reduced alertness seen in some rats during the early post-administration period could theoretically affect the von Frey test, reducing sensitivity. However, this effect is unlikely at the 6 h or later time-points, as we did not observe any sedative-related behaviours this long after drug administration. Interestingly, a significant increase in plantar sensitivity was seen in the IR-BUP group at 24 h. This was not observed with PO-BUP or SR-BUP administration, suggesting that factors such as the route of administration and the rate of drug delivery and distribution, influencing acute buprenorphine exposure, play a significant role and may be more influential on the observed effect than the nominal mg/kg dose administered. The non-opioid control analgesic, carprofen, did not alter sensitivity in the von Frey test at any time-point, consistent with the lack of effect of an anti-inflammatory drug in a non-injury model [24].

Earlier studies evaluating rodent depot SR-BUP formulations in rats have also reported significantly reduced sensitivity in the manual von Frey test when measured on an uninjured hind paw (contralateral to a surgically incised paw), however, they did not observe a significant effect following IR-BUP treatment [24, 25]. Conversely, a thermal stimulus (radiant heat) did not alter withdrawal responses in the uninjured paw. Similarly, Allen and Johnson evaluated the effect of buprenorphine in healthy rats using a thermal stimulus (radiant heat) on hind limb withdrawal latencies and observed only a transient effect at one hour [39]. The authors speculate that the type of test and the absence of injury may influence the ability to detect opioid effects. Together, these findings support the use of mechanical sensitivity tests, such as the von Frey assay, for evaluating opioid-induced modulations of sensory thresholds in uninjured animals, consistent with our results.

Assessing the effects of buprenorphine on sensory thresholds in a hypersensitive condition or in response to a painful stimulus may however allow for a more sensitive evaluation, as buprenorphine’s effects on sensory thresholds appear to be more pronounced in rats experiencing pain [24, 39, 40]. Not only the presence of pain or hypersensitivity, but also the type, intensity, and duration of the stimulus may influence the analgesic effect of buprenorphine, and the duration of effect should therefore be evaluated specifically for the given situation and animal species used to ensure sufficient analgesic coverage between re-applications [4, 28]. To address this, we evaluated the effect of Buvidal in a hypersensitive condition using a dural inflammation model of headache-like pain established in our lab [33], in which rats exhibit cephalic allodynia in the von Frey test. Using a hypersensitivity model rather than an overt pain model allows vehicle-treated controls to be included without experiencing significant pain. Cephalic (periorbital) sensitivity induced by dural inflammation was significantly reduced following SR-BUP treatment, with withdrawal thresholds well above baseline levels at the 3 h time-point. The effect persisted until the last time-point assessed (30 h), suggesting a more pronounced or prolonged effect of SR-BUP in hypersensitive tissue. In contrast, hind paw allodynia was not seen following dural inflammation, but withdrawal thresholds were still increased at the 3 h and 24 h time-points, indicating a systemic reduction in mechanical sensitivity, consistent with findings in pain-free animals.

In the present study, functional effects assessed by the von Frey test generally corresponded with systemic buprenorphine levels above the commonly cited 1 ng/mL threshold associated with analgesic activity. In the IR-BUP group, plasma concentrations exceeded this level at early time-points and paralleled the transient effects observed in naïve mechanical sensitivity. Plasma concentrations following SR-BUP administration were markedly higher than those observed after IR-BUP, reflecting both the higher administered dose and the slow-release kinetics of the depot formulation. In naïve animals, however, SR-BUP treatment did not produce similarly sustained changes in mechanical sensitivity at later time points. In contrast, the sustained systemic exposure of SR-BUP, remaining well above 1 ng/mL at the final sampling time-point (30 h), aligned with the prolonged behavioural effects observed in the hypersensitivity model, suggesting that both model sensitivity and the physiological state of the tissue influence the detectability of drug-induced changes. Previous studies investigating SR-BUP formulations in rats have reported differing plasma exposure profiles between males and females, potentially influenced by both rat strain and formulation characteristics; therefore, the present findings in male SD rats should not be directly extrapolated to females [41, 42]. Buvidal has an elimination half-life of approximately five days in humans, with peak levels typically reached about 24 h after subcutaneous administration [43]. Based on our data, peak exposure in rats also appears to occur within the first 24 h after injection. Others have observed that both IR-BUP and rodent SR-BUP formulations reach peak levels within 8 h in rats, suggesting that the human SR-BUP formulation may display a similarly early peak in rats.

The dose of SR-BUP used across the experiments in the present study (1.1–1.6 mg/kg) was slightly higher than the recommended doses for rodent long-acting buprenorphine formulations available in the United States (1.0–1.2 mg/kg [Buprenorphine Base Lab ER], 0.65 mg/kg [Ethiqa XR]). Correspondingly, systemic exposure observed here resulted in higher plasma concentrations than the peak levels of approximately 2–3 ng/mL reported within the first 24 h after dosing in male SD rats treated with rodent depot formulations [22, 25, 41]. These differences likely reflect both the somewhat higher nominal dose and the formulation-specific release kinetics of the human depot preparation. Despite the higher plasma concentration in this study, no severe adverse effects or injection site skin irritations were observed, although gross skin reactions have previously been reported with a rodent-specific slow-release formulation [22, 36]. Transient opioid-related behaviours, including pica-like chewing and reduced alertness, were observed shortly after treatment and are consistent with previously described buprenorphine-associated effects in rats [7, 23, 39]. Overall, the observed effects did not indicate severe or persistent adverse reactions under the conditions of the present study.

The high concentration and viscosity of the human SR-BUP formulation also present practical challenges for precise bodyweight-based dosing in rats, particularly when administering very small volumes using precision syringes. Consequently, some variability in the delivered dose between animals and across experiments is difficult to avoid. Therefore, the effective dose range needs to be wide enough to ensure both efficacy and safety. In the present study, a dose range of approximately 1.1–1.6 mg/kg Buvidal appeared effective and well tolerated, at least for a single injection, which for many applications would be sufficient. However, systematic evaluation of potential adverse effects, particularly following repeated dosing, is warranted prior to long-term use. In addition, the high concentration of the human formulation, combined with the practical limitations of administering very small volumes, may make rats weighing less than 300 g unsuitable for this treatment protocol due to an increased risk of overdosing. Notably, storage of an opened vial for up to three weeks did not detectably reduce efficacy in our model, supporting the feasibility of long-term use of a single Buvidal syringe.

Locomotor activity, as assessed by the OFT, was not significantly affected by buprenorphine treatment. However, a reduction in locomotor activity was observed between the first to the second test regardless of treatment, likely a result of reduced exploratory drive when the rats were presented to the test arena the second time. This effect was less apparent in the IR-BUP group, which also showed considerable inter-animal variability at the first time-point. Transient sedative-like behaviour observed in some buprenorphine-treated rats shortly after drug administration, although not systematically quantified, may have contributed to the observed variability and lower activity levels at the early OFT time-point. Such effects appeared less pronounced in SR-BUP-treated rats, consistent with the OFT findings. In line with this, Collins et al. did not observe sedation in SD rats following SR-BUP treatment, although a sedative effect was reported with transdermal buprenorphine [11].

One limitation of the present study is that only male rats were included. This approach was chosen to enable comparison with previous studies using males exclusively [22–25] and to avoid potential confounding effects of hormonal fluctuations associated with the oestrous cycle [44]. However, this limits the generalizability of the findings, as females may differ in pain sensitivity and analgesic response [45–47]. Additional studies are needed to evaluate the pharmacokinetics, efficacy, and tolerability of Buvidal in female rats to fully characterize its effects across sexes. Other limitations include the use of a single sensitivity test and evaluation in only one model of cephalic hypersensitivity. As a proof-of-concept study, the design was intentionally simplified to minimize experimental variables and focus on a single, well-characterized model. Finally, histological analysis of the injection site was not performed, and local tissue responses cannot be excluded, as inflammatory changes have been reported with rodent SR-BUP formulations [41].

## Conclusions

The human SR-BUP formulation Buvidal produced a prolonged reduction in mechanical sensitivity in male SD rats, with effects persisting beyond 24 h in a model of cephalic allodynia. These findings demonstrate sustained systemic exposure and associated behavioural effects consistent with the depot release properties of the formulation.

Within the scope of this exploratory study, Buvidal may represent a potential approach for achieving extended opioid effects in laboratory rats, particularly in settings where repeated dosing is challenging or rodent-specific slow-release formulations are not available. However, given that analgesic efficacy and tolerability may vary depending on strain, sex, analgesimetric test, and experimental model, further studies are required to evaluate these factors, as well as safety, and to address practical aspects of dosing and administration, before its use in analgesic regimens can be considered.

More broadly, the present findings highlight the potential of clinically approved depot formulations as tools for sustained drug delivery in preclinical research, warranting further investigation across a range of compounds and applications.

## Supplementary Information

Below is the link to the electronic supplementary material.

Supplementary Material 1

Supplementary Material 2

## Data Availability

Data will be made available upon request.

## Abbreviations

ANOVA: Analysis of variance
CAP: Carprofen
IR-BUP: Immediate-release buprenorphine
LLOQ: Lower limit of quantification
NaCl: Sodium chloride
OD: Optical density
OFT: Open field test
PO-BUP: Oral buprenorphine
p.o.: Per os
s.c.: subcutaneously
SD: Sprague Dawley
SR-BUP: Slow-release buprenorphine
ULOQ: Upper limit of quantification
%v/v: Volume/volume percent
