# Supplementary material for: Evaluation of a human slow-release buprenorphine formulation in rats – tactile sensitivity and plasma concentration
Source: Lab Anim Res. 2026 Aug 24;42:29. doi: 10.1186/s42826-026-00293-7 (PMC13501614; doi:10.1186/s42826-026-00293-7)
Supplement: Supplementary file 1 [file 42826_2026_293_MOESM1_ESM.docx]

**Supplementary materials**

**Animals**

Experiments were performed using a total of 108 male Sprague Dawley (SD) rats, 300-350 g (NTac:SD, Taconic Biosciences, Ejby, Denmark). The animals were group-housed (3-5 per cage) in Eurostandard Type IV cages (Tecniplast, Buguggiate, Italy) with dust-free wood granulate bedding and paper shreds nesting material (SAFE, Augy, France) under standard conditions with a 12-hour light/dark cycle, ambient temperature at 22 ± 2 °C, and a relative humidity of 55 %. Further enrichment consisted of red polycarbonate hut, tunnel, and hanging retreat, as well as aspen chew sticks, hay-cubes, and hemp ropes (Brogaarden, Lynge, Denmark). The animals had free access to standard pelleted chow (Safe A30, SAFE) and tap water ad libitum. Dried corn kernels, sunflower seeds, and peanuts (Brogaarden) were added in the cage feeder once or twice weekly. The rats were allowed to acclimatize for minimum seven days before study start.

The rats were maintained at a barrier facility free of the following pathogens:

| SDAV (Sialodacryoadenitis virus) | Clostridium piliforme | Giardia spp |
| --- | --- | --- |
| RMV (Rat minute virus) | Cryptosporidium spp | Spironucleus muris |
| MAD1 (Mouse adenovirus type 1) | Streptobacillus moniliformis | Entamoeba muris |
| MAD2 (Mouse adenovirus type 2) | Streptococcus pneumoniae | Tritrichomonas muris |
| RPV (Rat parvovirus) | Streptococcus sp. beta haemolytic Group A | Chilomastix sp |
| RTV (Rat theilovirus) | Streptococcus sp. beta haemolytic Group B | Helicobacter spp. |
| KRV (Kilham rat virus) | Streptococcus sp. beta haemolytic Group C | Helicobacter bilis |
| PVM (Pneumonia virus of mice) | Streptococcus sp. beta haemolytic Group G | Helicobacter ganmani |
| REO3 (Respiratory enteric virus III) | Salmonella spp. | Helicobacter hepaticus |
| Sendai | Pasteurella pneumotropica (Jawetz) | Helicobacter mastomyrinus |
| Hantaan | Pasteurella pneumotropica (Heyl) | Helicobacter rodentium |
| Toolan’s H-1 | Aspiculuris tetraptera | Helicobacter typhlonius |
| CAR bacillus | Syphacia obvelata | Myocoptes |
| Pneumocystis carinii | Syphacia muris | Myobia/Radfordia |
| Mycoplasma pulmonis |  |  |
