# Supplementary material for: Evaluation of a human slow-release buprenorphine formulation in rats – tactile sensitivity and plasma concentration
Source: Lab Anim Res. 2026 Aug 24;42:29. doi: 10.1186/s42826-026-00293-7 (PMC13501614; doi:10.1186/s42826-026-00293-7)
Supplement: Supplementary file 2 [file 42826_2026_293_MOESM2_ESM.docx]

**Suppl. Table S1. Results from statistical analyses presented in Fig.1 - 4.**

| **Figure** | **Groups analysed** | **Main test**  **F-values and p-values†** | **Post test**  **p-values** | **n/group** |
| --- | --- | --- | --- | --- |
| **Fig.1 -** **Tactile sensitivity following buprenorphine treatment** | | | | |
| Fig.1  Plantar thresholds |  | Repeated measures Mixed-effects model | Dunnett’s (Baseline vs post treatment) | |
|  | All five  treatment groups over  time | F_time_(4, 130)=40.48; p<0.0001 ****  F_treatment_(4, 55)=10.61; p<0.0001 ****  F_interaction_(16, 130)=7.25; p<0.0001 **** | Saline: 3h: 0.8337 (ns)  6h: 0.9620 (ns)  24h: 0.8240 (ns)  30h: 0.9982 (ns)  IR-BUP: 3h: 0.0031 **  6h: 0.0391 *  24h: 0.0264 *  30h: 0.9897 (ns)  PO-BUP: 3h: 0.0013 **  6h: 0.2173 (ns)  24h: 0.5696 (ns)  SR-BUP: 3h: 0.0006 ***  6h: 0.0002 ***  24h: 0.0580 (ns)  30h: 0.7608 (ns)  CAP: 3h: 0.9950 (ns)  6h: 0.7507 (ns)  24h: 0.7631 (ns)  30h: 0.8863 (ns) | 6-12  6-12  6-12  6-12  6-12 |
|  |  |  | Dunnett’s (Saline vs analgesic) |  |
|  |  |  | Baseline: IR-BUP: 0.8322 (ns) PO-BUP: 0.3908 (ns)  SR-BUP: 0.2634 (ns)  CAP: 0.7499 (ns)  3h: IR-BUP: 0.0152 * PO-BUP: 0.2875 (ns)  SR-BUP: 0.0012 **  CAP: 0.3964 (ns)  6h: IR-BUP: 0.0517 (ns) PO-BUP: 0.3176 (ns)  SR-BUP: 0.0001 ***  CAP: 0.9526 (ns)  24h: IR-BUP: 0.4238 (ns)  PO-BUP: 0.9992 (ns)  SR-BUP: 0.2017 (ns)  CAP: 0.8959 (ns)  30h: IR-BUP: >0.9999 (ns)  SR-BUP: 0.7041 (ns)  CAP: 0.6610 (ns) | 12-12  6-12  6  6-12  6-12 |

| **Fig.2 - Plasma buprenorphine concentration following a single subcutaneous administration** | | | | | |
| --- | --- | --- | --- | --- | --- |
| Fig.2  Plasma concentration |  | One sample t-test (Hypothetical value: 1) | | |  |
|  | IR-BUP | 3h: 0.0087 ** (Mean concentration: 1.890 ± 0.8439 ng/mL)  6h: 0.0240 * (Mean concentration: 1.520 ± 0.6070 ng/mL)  24h: <0.0001 **** (Mean concentration: 0.2700 ± 0.2058 ng/mL) | | | 10 |
|  | SR-BUP | 3h: 0.0002 *** (Mean concentration: 9.720 ± 4.711 ng/mL)  6h: <0.0001 **** (Mean concentration: 7.150 ± 2.174 ng/mL)  24h: <0.0001 **** (Mean concentration: 6.550 ± 1.440 ng/mL) | | | 10 |
| **Fig.3 - The effect of buprenorphine on locomotor activity** | | | | | |
| Fig.3.A  Distance travelled |  | Repeated measures two-way ANOVA | Sidak’s (20 min vs 24 h) | |  |
|  | All three treatment groups over time | F_treatment_(2, 17)=2.496; p=0.1121 (ns)  F_time_(1, 17)=11.65; p=0.0033 **  F_interaction_(2, 17)=1.225; p=0.3183 (ns) | Saline: 0.1584 (ns)  IR-BUP: 0.8012 (ns)  SR-BUP: 0.0228 * | | 6-7 |
| Fig.3.B  Time mobile |  | Repeated measures 2-way ANOVA | Sidak’s (20 min vs 24 h) | |  |
|  | All three  treatment groups over  time | F_treatment_(2, 17)=1.935; p=0.1750 (ns)  F_time_(1, 17)=17.42; p=0.0006 ***  F_interaction_(2, 17)=1.142; p=0.3425 (ns) | Saline: 0.1072 (ns)  IR-BUP: 0.4337 (ns)  SR-BUP: 0.0072 ** | | 6-7 |
| **Fig.4 - Tactile sensitivity following buprenorphine treatment in dural inflammation model** | | | | | |
| Fig.4.A  Periorbital thresholds |  | Repeated measures two-way ANOVA | | Dunnett’s (Pre-inj. vs baseline and post inj.) | |
|  | Saline  SR-BUP | F_treatment_(1, 14)=25.87; p=0.0002 ***  F_time_(2.775, 38.84)=51.53; p<0.0001 ****  F_interaction_(2.775, 38.84)=24.61; p<0.0001 **** | | Saline: Baseline: <0.0001 ****  3h: 0.9834 (ns)  24h: 0.4974 (ns)  30h: 0.5964 (ns)  SR-BUP: Baseline: <0.0001 ****  3h: <0.0001 ****  24h: 0.0191 *  30h: 0.0145 * | 8  8 |
|  | SR-BUP | Paired t-test | | |  |
|  |  | Baseline vs. 3h: 0.0018 ** | | | 8 |
| Fig.4.B  Plantar thresholds |  | Repeated measures two-way ANOVA | | Dunnett’s (Pre-inj. vs baseline and post inj.) | |
|  | Saline  SR-BUP | F_treatment_(1, 14)=15.27; p=0.0016 **  F_time_(3.292, 46.09)=18.52; p<0.0001 ****  F_interaction_(3.292, 46.09)=15.84; p<0.0001 **** | | Saline: Baseline: 0.3072 (ns)  3h: 0.5164 (ns)  24h: 0.6754 (ns)  30h: 0.8477 (ns)  SR-BUP: Baseline: 0.9687 (ns)  3h: <0.0001 ****  24h: 0.0465 *  30h: 0.9840 (ns) | 8  8 |
|  | SR-BUP | Paired t-test | | |  |
|  |  | Baseline vs. 3h: <0.0001 **** | | | 8 |

**Suppl. Table S1. continued**

CAP; carprofen, ns; non-significant (p≥ 0.05), IR-BUP; immediate-release buprenorphine, PO-BUP; oral buprenorphine, SR-BUP; slow-release buprenorphine.

*; p<0.05, **; p<0.01, ***; p <0.001, ****; p<0.0001

† Repeated-measures ANOVAs were performed with Geisser–Greenhouse correction applied where appropriate
